# Association of relative expression of *HSD11B-1* and *HSD11B-2* with pregnancy status in sheep

**DOI:** 10.1186/s12864-025-12186-5

**Published:** 2025-11-17

**Authors:** Numan Ullah, Mohamed Diaby, Nada N. A. M. Hassanine, Elsayed E. Hafez, Amr M. A. Rashad, Ahmed A. Saleh

**Affiliations:** 1 Faculty of Agriculture, Al-Azhar University, Cairo, 11651 Egypt; 2 Animal and Fish Production Department, Faculty of Agriculture (Al-Shatby), Alexandria University, Alexandria City, 11865 Egypt; 3 Plant Protection and Bimolecular Diagnosis Department, Arid Lands Cultivation Research Institute, City of Scientific Research and Technological Applications, New Borg Al Arab city, Alexandria 21934 Egypt

**Keywords:** Early pregnancy detection, HSD11B-1, HSD11B-2, Reproductive efficiency

## Abstract

**Background:**

Livestock profitability hinges on reproductive efficiency, which can be significantly affected by factors such as extreme weather conditions. Early and accurate pregnancy detection is vital for optimizing reproductive physiology and ensuring economic sustainability. This study employed quantitative Real-Time Polymerase Chain Reaction (qRT-PCR) to evaluate the expression levels of *HSD11B-1* and *HSD11B-2* genes in peripheral blood mononuclear cells (PBMCs) from five sheep breeds: Barki, Rahmani, Rahmani *x* Barki crossbred, Awassi, and Ossimi. A total of 500 ewes, with 100 from each breed, and 75 rams (15 from each breed) were selected for detailed analysis based on uniform age (21.00 ± 0.75 months) and weight (45.00 ± 3.00 kg). Blood samples were collected on specified days ranging from day 9 post-mating to prepartum luteolysis to analyze gene expression associated with different gestational stages.

**Results:**

The current analysis revealed significantly elevated levels of *HSD11B-1* and *HSD11B-2* gene expression in pregnant ewes compared to non-pregnant and control groups across all breeds. For the *HSD11B-1* gene, expression levels progressively increased and peaked between days 35 and 40 post-mating (*p* < 0.001), with all breeds showing significant fold-changes during this period. Rahmani *x* Barki crossbred demonstrated the highest fold-changes for this gene. Both *HSD11B-1* and *HSD11B-2* exhibited a significant increase from day 11 to day 18 post-mating (*p* < 0.01). However, their expression patterns differed during gestation. *HSD11B-1* recorded its highest expression levels (*p* < 0.001) during mid-gestation (days 35–45), while *HSD11B-2* peaked earlier (*p* < 0.001), particularly during the post-implantation phase (days 18–25).

**Conclusions:**

To the best of our knowledge, this study is the first to report a significant upregulation of both genes as early as day 12 post-mating, providing a new benchmark for early pregnancy detection, which previously focused on day 14. This insight into gene expression patterns provides valuable information for reproductive biology and may inform future breeding and management practices aimed at optimizing reproductive success in sheep.

**Supplementary Information:**

The online version contains supplementary material available at 10.1186/s12864-025-12186-5.

## Introduction

Livestock profitability fundamentally depends on reproductive efficiency. The fertility rate, which represents the percentage of births per thousand animals annually, typically reaches around 50% under optimal conditions [1–3]. Various factors, including extreme weather, can significantly impact fertility rates [4, 5]. Early detection of non-pregnant animals is vital for enhancing reproductive physiology [6, 7]. Identifying open females facilitates a more accurate assessment of conception rates and allows for the early detection of reproductive diseases [8–10]. Additionally, non-pregnant females can be re-bred, providing substantial economic advantages. Knowledge of pregnancy status is also crucial for making informed management decisions regarding nutrition and health care [11, 12]. Ultimately, improving reproductive efficiency not only boosts productivity but also ensures long-term economic sustainability [13–16].

Ultrasonography has emerged as a pivotal non-invasive technique for early pregnancy diagnosis in veterinary medicine, particularly due to its accuracy in detecting pregnancies as early as 25 to 30 days post-breeding. Methods such as ultrasonography enable early and accurate pregnancy detection; however, they have limitations. Early pregnancy diagnostics, particularly during the pre-implantation period, are inherently challenging, as the risk of embryonic mortality is notably elevated, complicating reproductive management and decision-making [17–19]. Understanding these implications is crucial for optimizing breeding practices and ensuring the health of both the embryo and the mother [20–22].

Key genes, Hydroxysteroid 11-beta dehydrogenase 1 (*HSD11B-1*) and *HSD11B-2*, play significant roles in pregnancy recognition and maintenance. These genes encode enzymes that catalyze the interconversion of cortisol and cortisone. *HSD11B-1* activates cortisone by converting it to cortisol, while *HSD11B-2* inactivates cortisol by converting it to cortisone [23–30]. Hormonal signals, including active cortisol, are integral to this process in sheep [31, 32]. Additionally, progesterone (P4) regulates gene expression and promotes conceptus elongation, further supporting successful pregnancy outcomes [33]. Understanding the intricate biochemical and hormonal pathways involved provides valuable insights into reproductive biology [34, 35] and informs strategies to enhance fertility and overall livestock productivity [35–37]. The expression of *HSD11B-1* and *HSD11B-2* is particularly important during the critical pre-implantation period for embryo implantation and survival. The induction of *HSD11B-1* and *HSD11B-2* expression in the endometrium is primarily driven by progesterone, with additional stimulation from interferon tau (IFNT) and prostaglandins secreted by the conceptus [23–28]. Through the action of *HSD11B-1*, the endometrium converts inactive cortisone into active cortisol, thereby facilitating conceptus development and implantation. Although conceptus-derived signals are localized within the uterus, the downstream molecular changes they induce such as altered gene expression in peripheral blood mononuclear cells (PBMCs) may be detectable in peripheral circulation. Thus, characterizing the expression of *HSD11B-1* and *HSD11B-2* in PBMCs during early pregnancy could provide a minimally invasive molecular marker for pregnancy detection in sheep [38].

Detecting the expression of these genes may assist in differentiating between open and pregnant sheep during the early stages of pregnancy (9 to 14 days post-mating) [23–28]. In sheep, parturition is triggered by elevated levels of fetal adrenal-derived cortisol, inducing a shift in placental steroidogenic activity from progesterone to increased estradiol production. This shift enhances the secretion of placental prostaglandin F2α, stimulating myometrial activity [39–41]. The luteolytic activity of cortisol-induced and placental-derived prostaglandin F2α is also significant in species where the corpus luteum partially contributes to progesterone production, such as pigs, cows, goats, mice, cats and rabbits [42].

Thus, the objectives of the present study were to: (1) investigate the potential for using *HSD11B-1* and *HSD11B-2* gene expression in PBMCs as an alternative approach for early pregnancy detection in sheep; and (2) measure *HSD11B-1* and *HSD11B-2* gene expression across various stages in five sheep breeds, including post-mating, post-implantation, mid-gestation, and prepartum luteolysis.

## Materials and methods

### Housing, caring and management

**Fig. 1:**
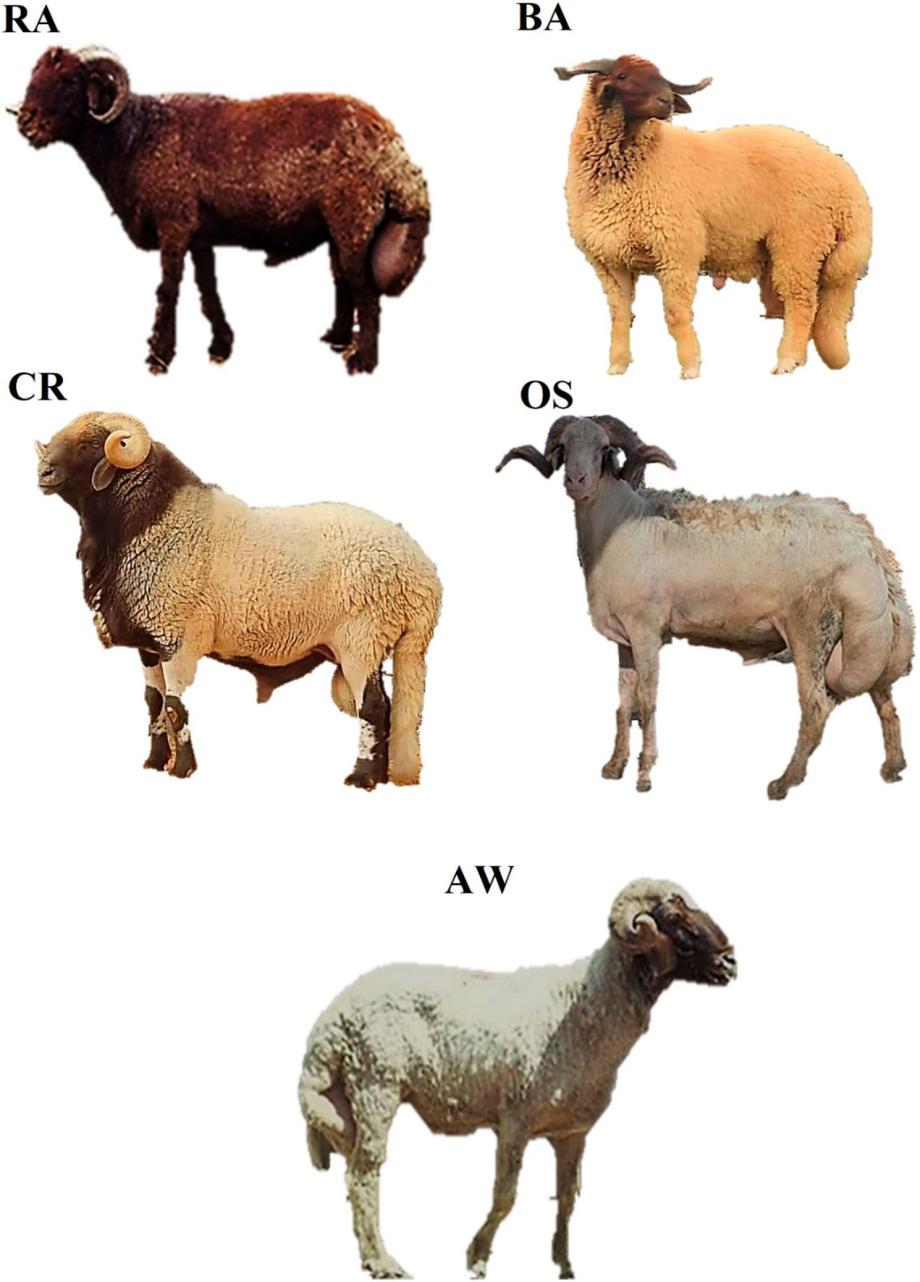
Sheep breeds scrutinized in the present study; Rahmani (RA), Barki (BA), Rahmani *x* Barki cross (CR), Ossimi (OS), and Awassi (AW)

The animal-related work in this study was overseen and approved by Alexandria University, Faculty of Agriculture (*Al-Shatby*), Egypt (No. AU082209203103) and Yangzhou University, College of Animal Science and Technology, China (No. YZU202501032). The experiment involved using several sheep breeds from a commercial dairy farm. Ewes and rams were housed separately in compartments. A total of 1044 non-pregnant healthy ewes were identified using ultrasound; Barki (*n* = 261♀), Rahmani (*n* = 189♀), Rahmani *x* Barki crossbred (*n* = 327 ♀), Awassi (*n* = 148♀), and Ossimi (*n* = 119♀) Fig. 1. The sheep breeds examined in this study originated from three distinct regions in Egypt: Alexandria city (coordinates: 31.206208°N, 29.919704°E), Sakha (coordinates: 31.087032°N, 30.948859°E), and the Matrouh governorate (coordinates: 31.336924°N, 27.205762°E). However, they were raised under a semi-intensive system at an animal farm known as the “experimental station” (coordinates: 31.206208°N, 29.919704°E). All tested animals were selected to be at the same age, chosen randomly from groups of contemporaries born within 1–2 weeks of each other.

After initial ultrasound screening, 100 ewes per breed (total *n* = 500 ewes) met inclusion criteria (age: 21.00 ± 0.75 months; weight: 45.00 ± 3.00 kg). Seventy-five fertile rams (15 per breed) were included for mating. To establish controls, 30 ewes per breed (150 total) were isolated from hormonal treatments. The remaining 70 ewes per breed (350 total) were then randomly selected to receive estrous synchronization. This involved a standard protocol using prostaglandin F2α (PGF2α) and gonadotropin-releasing hormone (GnRH). Each ewe received two 0.5 ml intramuscular injections of cloprostenol sodium (a synthetic PGF2α analogue; 125 µg per dose; cyclomate, Thermo Fisher Scientific, USA), administered 7 days apart. This was followed by a single 0.5 ml intramuscular injection of gonadotropin-releasing hormone (GnRH) (100 µg of gonadorelin hydrochloride; Cystorelin, Boehringer Ingelheim, USA) 30 h after the final cloprostenol injection. PGF2α analogue induces luteolysis, synchronizing the regression of the corpus luteum, while the subsequent GnRH injection synchronizes the ovulation of the resulting follicular wave. The protocol was applied according to Kal et al. [43] and Silva et al. [44] with some modifications.

Estrous behaviour was confirmed in all 350 ewes using vasectomized teaser rams at a ratio of 1:20–25 (one ram per 20 to 25 ewes). Receptivity was assessed through direct observation for standing heat during two 30-minute sessions daily (at 08:00 and 16:00 h). The ewe was considered in estrus upon displaying a standing reflex when mounted by a teaser ram. Following confirmation of estrus, mating with fertile, breed-matched rams (15 rams per breed) was allowed for 48-hour period, after which all rams were removed. Control ewes were housed separately under the same environmental conditions (feed, water, space, light, and temperature) as the experimental groups but were maintained in dedicated pens to prevent any physical or visual contact with rams throughout the experiment.

### Mating and sampling

Fifteen fertile rams per breed were introduced for a 48-hour mating period with the estrous-induced ewes (*n* = 70/breed). The remaining ewes (*n* = 30/breed) were maintained as a non-mated control group **(**Table 1**)**. The first day of mating was designated day 0 of conception.

**Table 1:** Overview of experimental groups in the studied sheep breeds

Tested Animals						Ultrasound test on day 30		Randomly Selected Animals for Genetic Investigation				
Breeds		Ewes			Rams							
	RSA-ASA^a^		TEW^b^	CL^c^	------	PRG^d^	NPRG^e^	CL^c^	PRG^d^		NPRG^f^	
	No.		No.	No.	No.	No.	No.	Pools (Pools x samples		Pools		Pools
Barki	100		70	30	15	54	16	5 × 6		6 × 6		3 × 3
Rahmani	100		70	30	15	61	9	5 × 6		6 × 6		3 × 3
CR^f^	100		70	30	15	64	6	5 × 6		6 × 6		3 × 2
Ossimi	100		70	30	15	59	11	5 × 6		6 × 6		3 × 3
Awassi	100		70	30	15	58	12	5 × 6		6 × 6		3 × 3
Total	500		350	150	75	296	54	150		180		42

^a^RSA-ASA Randomly selected Animals at the same age (21.00±0.75 months) and weight (45.00±3.00 kg) to measure the expression of *HSD11B-1* and *HSD11B-2* genes during the period of 9 to 40 days post-conception and prepartum luteolysis

^b^TEW Randomly selected ewes that allowed to mate with rams

^c^CL Control/Randomly selected ewes that not allowed to mate with rams

^d^PRG Pregnant

^e^NPRG non-Pregnant

^f^CR Rahmani *x* Barki crossbred

Blood samples (2 × 2 ml) were collected from the jugular vein of each tested sheep using sterile blood collection tubes (vacutainers), with 0.5 ml of 2.7% EDTA for preservation [1] at defined gestational stages: post-mating (days 9, 10, 11, 12, 13, 14 and 16), post-implantation (days 18–25), mid-gestation (days 35–40) and prepartum luteolysis. The timing for prepartum luteolysis sampling was estimated based on the known average gestation length of 147 days for the studied sheep breeds. Samples were collected approximately 2–3 days before the expected due date, a period associated with the initiation of the luteolytic process. Collected blood samples were immediately stored at 4 °C and processed for PBMC isolation within 2 h of collection to ensure cell viability and RNA integrity.

To account for individual biological variability while optimizing statistical power and feasibility, a pooling strategy was employed for RNA extraction and qRT-PCR analysis [45, 46]. For each specific examination day within a stage (e.g., day 18, day 20, etc.), PBMCs were isolated from individual ewes sampled on a specific day. Subsequently, equal masses of total RNA (500 ng) from multiple ewes of the same breed, pregnancy status, and sampling day were combined to form a single pooled sample. This approach enhances the detection of consistent group-level expression patterns by averaging out individual variations. The number of ewes per pool differed between groups to pragmatically accommodate the available number of animals; (a) Control groups (non-mated): 5 pools per breed (each from 6 ewes), (b) pregnant groups: 6 pools per breed (each from 6 ewes), (c) non-pregnant groups (mated group): 3 pools per breed (each from 3 ewes), except for the Rahmani *x* Barki crossbred (2 pools from 3 ewes each). Critically, samples from different examination days were processed and analyzed separately; no mixing of samples across days occurred. This approach ensured precise resolution of temporal gene expression dynamics.

Pregnancy was diagnosed using the specific gene expression markers *HSD11B-1* and *HSD11B-2* from days 9 to 40 and prepartum luteolysis, and was subsequently confirmed via transrectal ultrasonography on day 30 post-mating using transrectal ultrasonography. This was performed using a portable B-mode ultrasound scanner (E.I. Medical Imaging IBEX^®^ Lite, Loveland, CO, USA) equipped with a linear rectal transducer (Model EIL8-RFAB, 8.0 MHz). The procedure was conducted by a trained veterinarian following standard aseptic protocols.

The study focused on days 9 to 40 post-mating and prepartum luteolysis, a critical pre-implantation period in sheep when ultrasound cannot detect pregnancy due to the absence of visible embryonic structures, aiming to explore the potential of monitoring *HSD11B-1* and *HSD11B-2* gene expression in PBMCs as an alternative method for early pregnancy detection in livestock. Also, measuring *HSD11B-1* and *HSD11B-2* genes` expression for five sheep breeds during; post-mating (days; 9, 10, 11, 12, 13, 14 and 16), post-implantation (days 18–25), mid-gestation (days 35–40) and prepartum luteolysis, and investigate the differentiation among several sheep breeds.

### Isolation of peripheral blood mononuclear cells (PBMCs) and total RNA

PBMCs were isolated from the collected blood samples of each examined animal, according to Kleiveland et al. [47]. While, the protocol’s Total RNA isolation steps were implemented in accordance with standard procedures, following the method of Haq et al. [48]. The total RNA was quantified using a spectrophotometer (Qubit™ Fluorometer, Thermo Scientific™, USA) to assess the concentration and purity of the RNA for qRT-PCR. A volume of 1 µL from each sample was utilized to determine the quantity and purity of the isolated RNA. The RNA purity, as indicated by the A260/280 ratio, was recorded between 1.7 and 2.0, and the concentration of all samples was above 200 ng/µL. To eliminate potential genomic DNA contamination, each sample underwent treatment with the RQ1 RNA-Free DNase kit (Promega, Dübendorf, Switzerland).

### Molecular assessments and gene expression

Quantitative Real-Time Polymerase Chain Reaction (qRT-PCR) was employed to assess the relative expression levels of *HSD11B-1* and *HSD11B-2*, utilizing primers previously validated by Simmons et al. [23]. based on the ovine *HSD11B-1* sequence (GenBank accession no. NM_001009395) and the ovine *HSD11B-2* sequence (GenBank accession no. NM_001009460.1), which plays a crucial role in mediating pregnancy. The reference gene, Beta-Actin (*ACTB*), was validated in sheep by Sahu et al. [49]., as presented in Table 2. The expression fold changes for *HSD11B-1* and *HSD11B-2* were normalized using *ACTB* as an internal standard.

**Table 2:** The oligonucleotide primer pairs used for quantitative qRT-PCR analysis include both tested genes and housekeeping control genes

Tested Genes									
Primer Symbol	**Accession No.**			**Primer Sequence (**5’−3’)		**Amplicon Size**		***Ref.***	
*HSD11B-1*	NM_001009395			5’-CATTCTGGG GATCTTCTTGG-3’		534 bp		Simmons et al. [23]	
				5’-GAATAGGCAGCAGCAAGTGG-3’					
*HSD11B-2*	NM_001009460.1			5’-AGTTCACCAAGGTCCACACC-3’		457 bp			
				5’-TGCTCGATGTAGTCCTCACC-3’					
Reference Genes/housekeeping controls									

Gene Symbol		**Accession No.**	**Primer Sequence (**5’−3’)		**Amplicon Size**		**Efficiency**		***Ref.***
*ACTB*		NM_001314342.1	5’-CTCTTCCAGCCT TCCTTCCT-3’		101 bp		102.77		Sahu et al. [49]
			5’-TAAAGGTCCTTG CGG ATG TC-3’						

Complementary DNA (cDNA) was synthesized from 500 ng of total RNA using the TO-Preal™ qRT-PCR kit (Enzynomics, Daejeon, South Korea). The qRT-PCR kit is designed for one-step qRT-PCR, where both cDNA synthesis and PCR amplification occur sequentially in a single tube. It integrates reverse transcriptase and hot-start DNA polymerase in a single enzyme mix, and was used according to the manufacturer’s instructions. Briefly, the reverse transcription reaction was carried out at 50 °C for 30 min.

The reaction mixture comprised a total volume of 20 µl, which included 10 µl of SYBR Green qPCR Master Mix, 1 µl of forward primer, 1 µl of reverse primer, 1 µl of the synthesized cDNA (equivalent to 25 ng of original RNA template), and distilled deionized water (ddH₂O) to bring the volume to 20 µl.

The amplification program included an initial hold at 50 °C for 30 min, followed by an initial denaturation step at 95 °C for 10 min. This was followed by 45 cycles of denaturation at 95 °C for 5 s, and annealing/elongation at 61.2 °C for 30 s for *HSD11B-1* and at 60.5 °C for 30 s for *HSD11B-2.*

Gene expression levels were measured using SYBR Green-based real-time qPCR on an ABI PRISM 7500 Sequence Detection System (Applied Biosystems). The specificity of amplification was confirmed by melt curve analysis following the amplification cycles [45, 50].

The relative expression ratio was accurately quantified and calculated according to Livak and Schmittgen [51]. Accordingly, for each biological sample, the difference (Δ) in quantification cycle value (C_T_) between the target (C_T (target)_) averaged from several technical repeats) and the reference (C_T (reference)_), a fixed C_T_ value was used for all samples) was first transformed into relative quantities (RQ) using the exponential function with the efficiency (E) of the PCR reaction.

The C_T_ (threshold of cycle) value of each detected gene was determined by automated threshold analysis on ABI System. The C_T_ value of each target gene was normalized to C_T (reference)_ to obtain ΔC_T (target)_ where.

$$\triangle C_{T\left(target\right)}=\left(C_{T\left(target\right)}-C_{T\left(reference\right)}\right),$$

$$\triangle C_{T\left(control\right)}=\left(C_{T\left(control\right)}-C_{T\left(reference\right)}\right),$$

The relative expression quantity of the target gene was indicated with ΔΔCT = (ΔC_T (target)_ – ΔC_T (control)_) according to 2^−ΔΔCt^ algorithm.

### Statistical analysis

All quantitative data on relative gene expression were analyzed for arithmetic means and standard errors using SPSS version 20.0 at various time points after conception. An analysis of variance (ANOVA) was conducted in SPSS to calculate the p-value, enabling the estimation of differences between the different time points. Additionally, the Least Significant Difference (LSD) test at the 0.05 significance level (LSD₀.₀₅) was performed to compare gene expression levels at each time point with one another. This comprehensive statistical analysis ensures the robustness and reliability of the findings, facilitating a deeper understanding of the temporal dynamics in gene expression across different stages of pregnancy.

## Results

### Gene expression during the period of 9 to 40 days post-conception

**Fig. 2:**
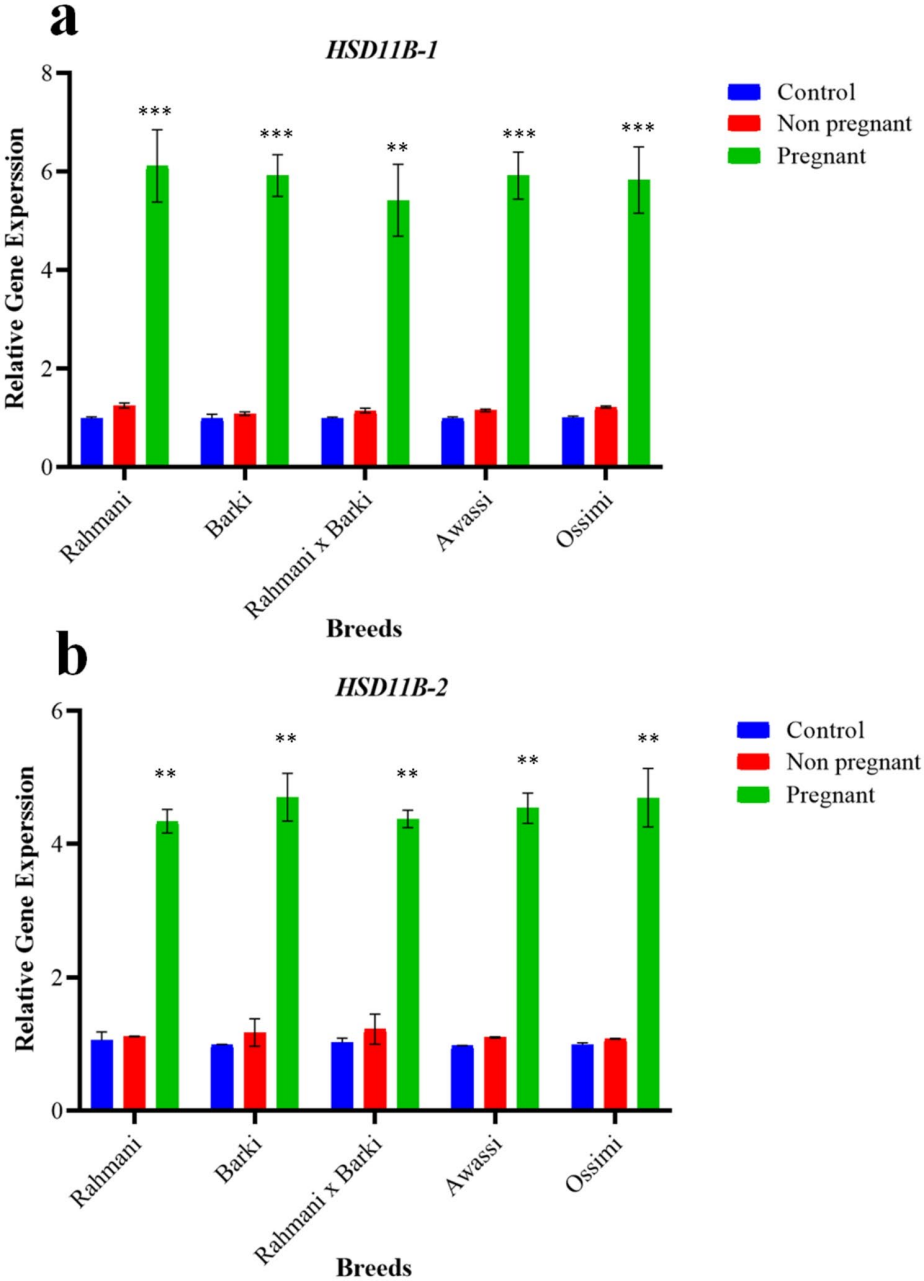
The overall dynamic changes in relative RNA expression of (**A**) 11-*beta*-hydroxysteroid dehydrogenase type-1 (*HSD11B-1*) and (**B**) 11-*beta*-hydroxysteroid dehydrogenase type-2 (*HSD11B-2*) gene, with reference gene (*ACTB)* across the specified timeframe (day 9 to day 40) in distinct groups of sheep; Rahmani, Barki, Rahmani *x* Barki crossbred, Awassi and Ossimi. The groups under observation include a control group/non-mated ewes (*n* = 150), pregnant ewes (*n* = 180) and non-pregnant ewes/mated ewes (*n* = 42). Results are shown as mean ± SEM, with statistical significance denoted by **p < 0.01 and ***p < 0.001 compared to the control group

The relative expression of *HSD11B-1* and *HSD11B-2* genes was analyzed compared to the housekeeping control gene in different groups of sheep from days 9 to 40 post-conception to investigate their potential involvement in pregnancy. The choice of using *ACTB* as a reference gene was based on its known stability and constant expression levels [52, 53]. Expression results from days 9 to 40 post-conception, along with an ultrasound assessment conducted on day 30, indicated that the study groups included 64 pregnant ewes and 6 non-pregnant ewes of Rahmani *x* Barki crossbred. In contrast, Barki breed comprised 54 pregnant ewes and 16 non-pregnant ewes **(**Table 1**)**. Figure 2 presents a visual representation of the overall period from day 9 to post-conception regarding the relative expression of the *HSD11B-1* **(**Fig. 2A**)** and *HSD11B-2* **(**Fig. 2B**)** genes in various sheep groups. It highlights the differences observed throughout this timeframe (from day 9 to day 40 post-conception), particularly emphasizing a significant superiority in the pregnant group (*p* < 0.001).

### Gene expression patterns

#### HSD11B-1 gene

**Fig. 3:**
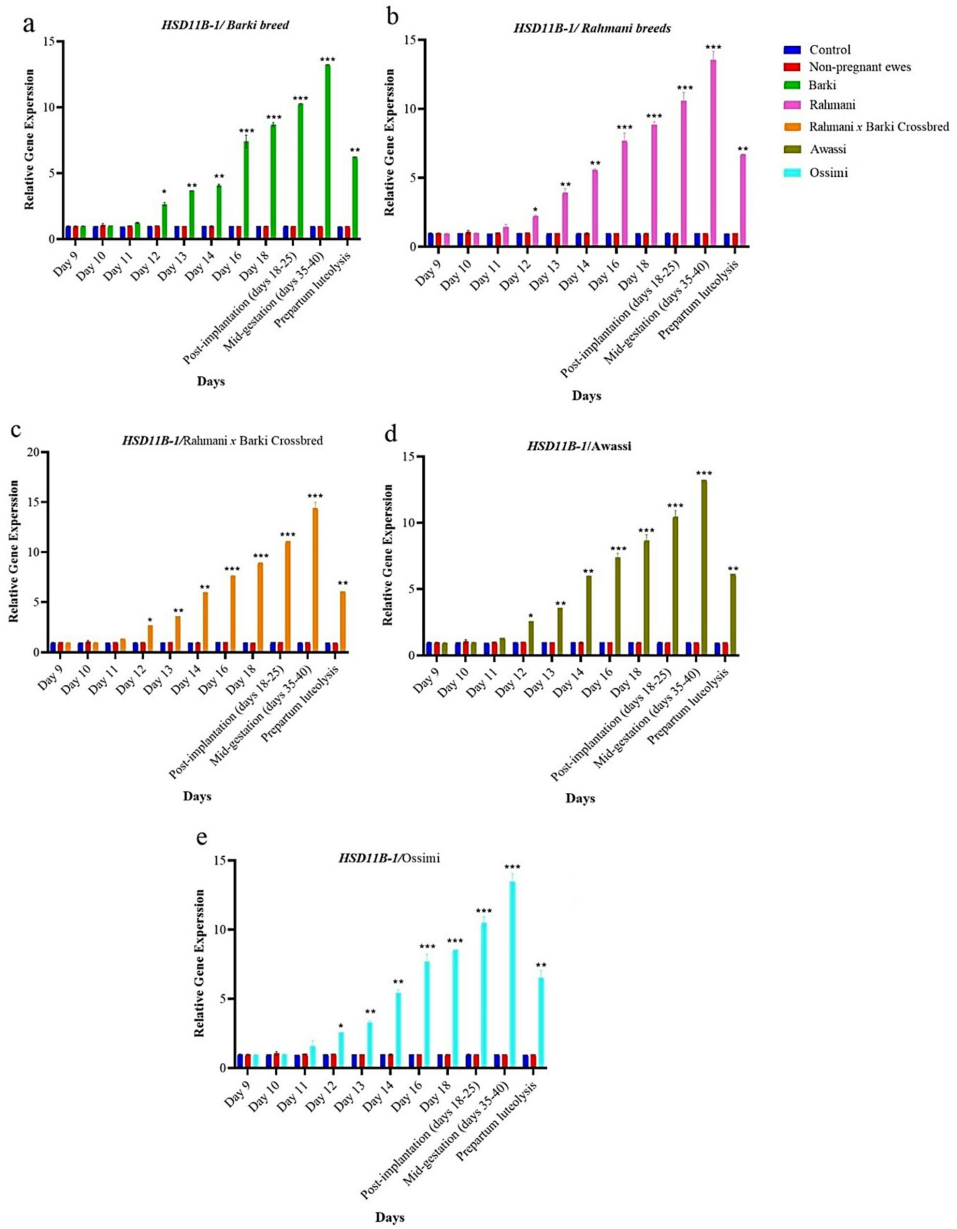
The dynamic changes in relative RNA expression of 11-beta-hydroxysteroid dehydrogenase type-1 (*HSD11B-1*) gene with housekeeping control gene on days 9 to 40 post-mating and prepartum luteolysis in distinct groups of sheep; (**a**) Barki, (**b**) Rahmani, (**c**) Rahmani *x* Barki crossbred, (**d**) Awassi and (**e**) Ossimi. The groups under observation include a control group/non-mated ewes (*n* = 150), pregnant ewes (*n* = 180) and non-pregnant ewes/mated ewes (*n* = 42). Results are shown as mean ± SEM, with statistical significance denoted by *p < 0.05, **p < 0.01 and ***p < 0.001 compared to the control group

The expression levels of *HSD11B-1* gene were significantly higher in pregnant subjects compared to non-pregnant and control groups across all breeds studied. This elevation in gene expression suggests a potential role for this gene in early pregnancy detection. The results demonstrated that pregnant groups exhibited notably increased expression levels throughout the entire assessment period. Specifically, Rahmani breed showed fold changes increasing from 0.95 at the beginning of the period to 13.95 by mid-gestation (day 40). Barki breed experienced fold changes rising from 0.99 to 13.24 over the same timeframe. Rahmani *x* Barki crossbred displayed the highest levels of gene expression, with an increase from 0.95 to 15.08. Meanwhile, the Awassi breed exhibited fold changes from 0.94 to 13.23, and the Ossimi breed showed an increase from 0.91 to 14.13 **(**Fig. 3**)**.

#### HSD11B-2 gene

**Fig. 4:**
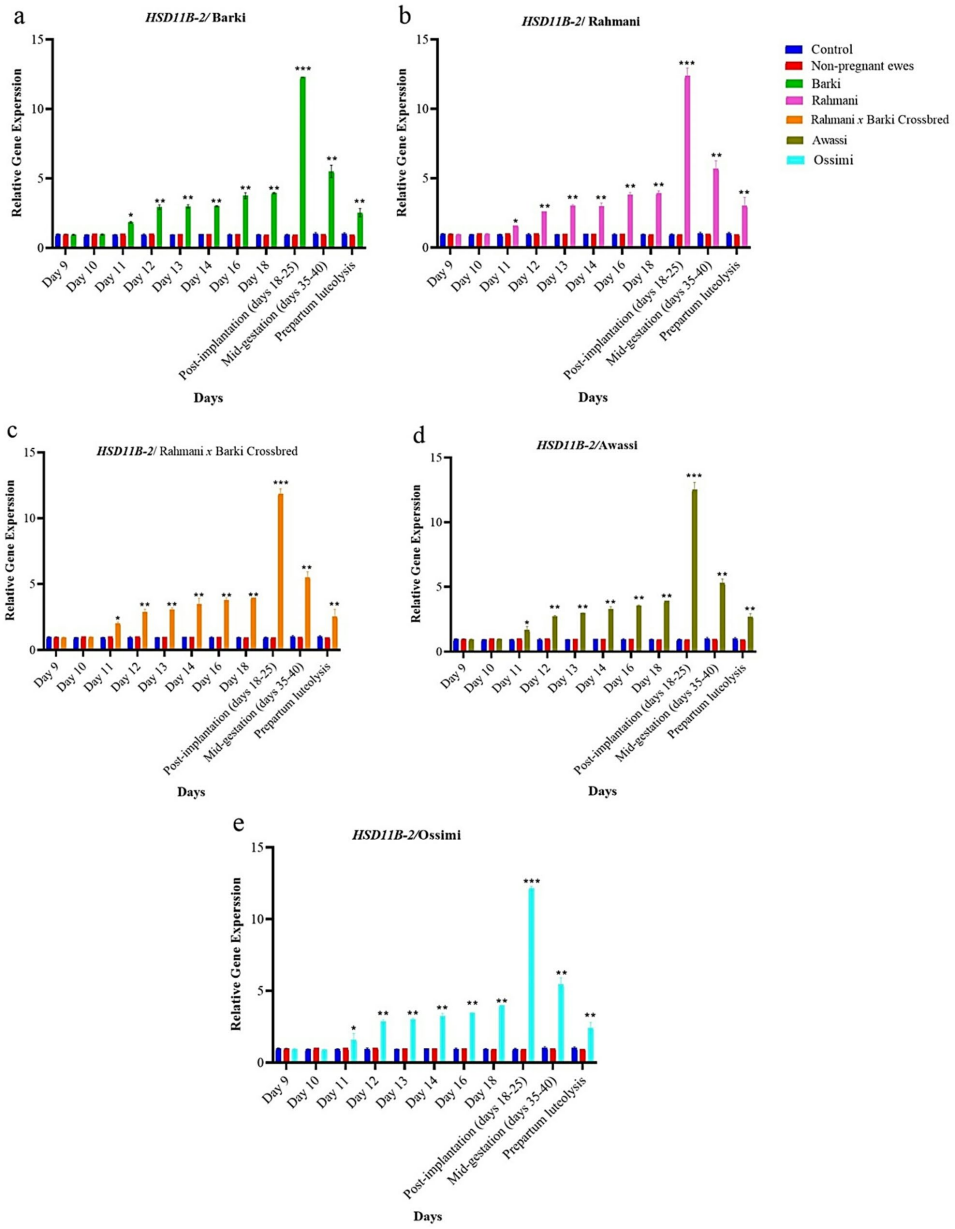
The dynamic changes in relative RNA expression of 11-beta-hydroxysteroid dehydrogenase type-2 (*HSD11B-2*) gene with housekeeping control genes on days on days 9 to 40 post-mating and prepartum luteolysis in distinct groups of sheep; (**a**) Barki, (**b**) Rahmani, (**c**) Rahmani *x* Barki crossbred, (**d**) Awassi and (**e**) Ossimi. The groups under observation include a control group/non-mated ewes (*n* = 150), pregnant ewes (*n* = 180) and non-pregnant ewes/mated ewes (*n* = 42). Results are shown as mean ± SEM, with statistical significance denoted by *p < 0.05, **p < 0.01 and ***p < 0.001 compared to the control group

Similar patterns were observed for the expression of *HSD11B-2* gene throughout the entire assessment period. Within pregnant groups, Rahmani breed’s fold changes rose from 0.95 at the beginning of the period to 5.35 by mid-gestation (day 40). Barki breed’s expression levels increased from 0.94 to 5.26. Rahmani *x* Barki crossbred exhibited high expression levels, with fold changes increasing from 0.94 to 5.36. Awassi breed demonstrated an increase from 0.91 to 5.12, while Ossimi breed’s fold changes rose from 0.99 to 5.21 **(**Fig. 4**)**.

### Early pregnancy detection and gene expression on days 11 to 18 in the studied sheep breeds

It is worth noting that a significant increase in *HSD11B-1* and *HSD11B-2* expression can be observed from days 11 to 18. According to our data, day 12 presents a significant increase in the fold change of both *HSD11B-1* **(**Fig. 3**)** and *HSD11B-2* **(**Fig. 4**)** genes across all experimental groups compared to the control group. This pronounced expression suggests that gene expression levels can serve as early indicators of pregnancy as early as day 12, preceding the previously reported day 14 benchmark [23].

The current findings indicate that Rahmani *x* Barki crossbred exhibited the highest peak expression levels of both *HSD11B-1* and *HSD11B-2* genes among all the studied breeds. For *HSD11B-1*, Rahmani displayed a peak fold-change of 2.28 on day 12, with the fold-change ranging from 2.55 in Ossimi to 2.67 in Rahmani *x* Barki crossbred **(**Fig. 3**)**. For *HSD11B-2*, the calculated fold-change for Rahmani on day 12 was 2.59, ranging from 2.29 in Ossimi to 2.89 in Rahmani *x* Barki crossbred **(**Fig. 4**)**.

This significant upregulation suggests a robust physiological response linked to early pregnancy detection. The elevated gene expression in Rahmani and its hybrids underscores their distinctive metabolic and physiological adaptations, making these breeds particularly suitable for early pregnancy detection programs aimed at enhancing reproductive efficiency and productivity (Figs. 3 and 4).

### Consistent upregulation patterns

Barki and Awassi breeds exhibited consistent patterns of upregulation for both genes, underscoring their importance in the early detection of pregnancy. Barki breed reached its highest expression level for *HSD11B-1* on the 12th day, with expression levels observed in various crossbred groups. For *HSD11B-2*, Barki breed similarly achieved its peak expression on the same day, with different levels seen in Ossimi and Rahmani *x* Barki crossbreeds. Awassi breed also showcased significant expression levels for both genes on the 12th day (Figs. 3 and 4).

These significant expression levels, although slightly lower compared to Rahmani breeds, indicate strong metabolic pathways influenced by *HSD11B-1* and *HSD11B-2* genes. The uniform increase and decrease in expression levels over time reflect stable regulatory mechanisms, ensuring predictable physiological responses crucial for early pregnancy detection and effective reproductive management. Ossimi breed displayed distinctive trends, peaking at 2.55 for *HSD11B-1* and 2.29 for *HSD11B-2* on day 12 **(**Figs. 3 and 4**)**.

Although following a similar temporal pattern to other breeds, the gene expression levels in Ossimi were generally lower. This divergence suggests that Ossimi may possess different regulatory or metabolic pathways affecting *HSD11B-1* and *HSD11B-2* expression. These lower responsiveness levels might be attributable to unique genetic characteristics or specific environmental adaptations.

### Pregnancy and gene expression on days 13, 14, 16 and 18 in the studied sheep breeds

During the observation period from the 13th to 18th days, both *HSD11B-1* and *HSD11B-2* genes exhibited marked fluctuations in expression across different breeds, highlighting their critical role in early pregnancy detection. Notably, *HSD11B-1* gene demonstrated a significant increase in expression, particularly in Awassi breed, with fold changes reaching approximately 3.68 on the 13th day and peaking at around 4.02 on the 14th day, accompanied by a *p*-value of 0.001, indicating strong statistical significance. This trend illustrates the inherent responsiveness of *HSD11B-1* gene expression in relation to pregnancy status.

Conversely, *HSD11B-2* also displayed a dynamic response during the same period, with fold changes peaking at about 3.03 on the 14th day. However, a slight decline was observed in its expression by the 18th day in certain groups. The 16th and 18th days further revealed distinct expression patterns between the two genes. On the 16th day, *HSD11B-1* reached impressive levels with fold changes nearing 8.0, indicating pronounced responsiveness as pregnancy advances, while *HSD11B-2* exhibited more moderated expression, showing fold changes around 3.63.

By the 18th day, a notable divergence emerged, with *HSD11B-1* continuing to demonstrate elevated expression, with fold changes approaching 8.79, reaffirming its role as a robust indicator of pregnancy progression. In contrast, *HSD11B-2* showed a slight decline in expression, with fold changes at approximately 3.92, suggesting a potential stabilization in hormone regulation as gestation advances **(**Figs. 3 and 4**)**.

These observed differences indicate that while both genes are crucial for early pregnancy detection, *HSD11B-1* acts as a more dynamic marker responsive to changes in pregnancy status, whereas *HSD11B-2* likely plays a complementary role. The interaction of expression patterns across various breeds suggests the influence of breed genetics and reproductive status, emphasizing the potential of utilizing these genes in conjunction for more accurate early pregnancy diagnostics in ewes.

### Pregnancy and gene expression on days 18 to 25 in the studied sheep breeds

During the post-implantation period, spanning from days 18 to 25 of pregnancy, the expression levels of *HSD11B-1* and *HSD11B-2* genes exhibit notable differences, highlighting their distinct functional roles. The expression of *HSD11B-1* gene is markedly elevated in pregnant subjects across all studied breeds, suggesting an upregulatory role in the establishment and maintenance of pregnancy. Notably, Rahmani *x* Barki crossbred shows a significant increase in *HSD11B-1* expression, reaching levels of approximately 11.08-fold compared to the control group, with the fold-change ranging from about 10.18-fold in Awassi breed to 11.08-fold in Rahmani *x* Barki crossbred. Barki breed also exhibited a substantial increase, reaching approximately 10.69-fold.

On the other hand, the expression of *HSD11B-2* gene follows a similarly high pattern during the same period, with fold changes ranging from approximately 12.08-fold in Rahmani *x* Barki crossbred to 12.69-fold in Barki breed **(**Figs. 3 and 4**)**. While both *HSD11B-1* and *HSD11B-2* show parallel expression patterns, their distinct roles suggest a complementary mechanism during pregnancy. Specifically, while *HSD11B-1* acts as a dynamic marker that supports the hormonal environment essential for pregnancy progression, *HSD11B-2* functions primarily as a regulatory mechanism that counterbalances elevated cortisol levels to protect the developing fetus. Elevated *HSD11B-1* expression appears to maintain a cortisol-rich environment crucial for fetal development and maternal adaptation. Meanwhile, the sustained elevated expression of *HSD11B-2*, which facilitates the conversion of active cortisol to inactive cortisone, indicates a protective mechanism against excessive glucocorticoid exposure to the fetus. This differential gene expression underscores the complexity of hormonal regulation that is essential for a successful pregnancy.

### Mid-gestation (days 35–40)

During the mid-gestation period, covering days 35 to 40, elevated expression levels of *HSD11B-1* and *HSD11B-2* genes continue to be observed across all studied breeds. *HSD11B-1* expression remains high, ranging from approximately 13.14-fold in Ossimi breed to 14.09-fold in Rahmani x Barki crossbred, emphasizing the sustained importance of this gene in ongoing gestational processes. In contrast, *HSD11B-2* expression shows a decrease, with levels observed at around 5.12-fold in Awassi breed and 5.35-fold in Barki breed **(**Figs. 3 and 4**)**.

During this period, the maternal physiological system stabilizes to support continued fetal growth. The persistently high expression levels of *HSD11B-1*, combined with the decreased but still significant levels of *HSD11B-2*, suggest that both genes likely play crucial roles in maintaining the conditions necessary for successful fetal development and maternal health. Specifically, the sustained elevated levels of *HSD11B-1* highlight its role in supporting the hormonal environment essential for fetal growth, while the moderated expression of *HSD11B-2* may reflect a balancing mechanism that helps regulate cortisol levels.

### Post-implantation and mid-gestation

During the post-implantation period, spanning days 18 to 25, *HSD11B-1* expression levels are significantly elevated across all pregnant sheep breeds, with notable values in Awassi and Rahmani *x* Barki crossbred. During late gestation (days 18–25), *HSD11B-2* expression levels exceed those of *HSD11B-1*, suggesting a heightened regulatory role for *HSD11B-2* during this period, particularly in the Rahmani *x* Barki and Barki breeds. However, in mid-gestation, *HSD11B-1* expression is significantly higher **(**Figs. 3 and 4**)**.

As the gestation progresses into the mid-gestation phase, encompassing days 35 to 40, *HSD11B-1* expression continues to rise, emphasizing its critical contribution to maintaining pregnancy. This upward trend is particularly pronounced in Rahmani *x* Barki crossbred. Conversely, the expression of *HSD11B-2* sees a decline, suggesting a reduced necessity for cortisol regulation as pregnancy advances, with lower levels observed in breeds such as Awassi and Barki.

### Prepartum luteolysis

Prepartum luteolysis occurs toward the end of gestation and involves the regression of the corpus luteum, leading to a decrease in progesterone levels and preparing the body for parturition. The expression levels of *HSD11B-1* and *HSD11B-2* in earlier stages suggest their potential involvement in the hormonal transitions necessary for lambing. Although specific expression levels during prepartum luteolysis are not detailed, previously observed high levels imply an ongoing role in the process.

The differential expression of *HSD11B-1* and *HSD11B-2* genes across various breeds underscores their significance in early pregnancy detection in sheep. Specifically, *HSD11B-1* expression levels during prepartum luteolysis range from approximately 6.09-fold in Rahmani *x* Barki crossbred to about 6.72-fold in Barki breed. Rahmani breed and its crossbred variant exhibit the highest responsiveness, indicating superior physiological adaptations for early pregnancy detection. For *HSD11B-2*, expression levels during this phase range from approximately 2.69-fold in Ossimi breed to about 2.71-fold in Rahmani breed. Worth mentioning, Fig. S1 represents the pure breeds and their progeny included in the present study seven months postpartum.

The consistent upregulation of *HSD11B-1* and *HSD11B-2* in Barki and Awassi breeds, along with unique expression patterns in Ossimi breed, provides valuable insights for enhancing reproductive management and breeding strategies across these genetic lines.

## Discussion

In the present study, we analyzed *HSD11B-1* and *HSD11B-2* gene expression in PBMCs of pregnant sheep from five different breeds. The significant and early upregulation of both genes in pregnant ewes compared to non-pregnant and control groups, detectable as early as day 12 post-mating, reveals their potential as novel biomarkers for early pregnancy detection and underscores their involvement in early gestational processes.

The significant upregulation of *HSD11B-1* and *HSD11B-2* in PBMCs during early pregnancy aligns with their established roles in the endometrium. Expression levels for the *HSD11B-1* gene progressively rose after Day 11, reaching their most notable peak between days 35 and 40. While, *HSD11B-2* expression peaked earlier, particularly between days 18 and 25, and then declined toward parturition. Although the overall expression pattern was consistent across all studied breeds, the magnitude of expression varied between them. Our findings in circulating immune cells corroborate the work of Simmons et al. [23]., who demonstrated that in the ovine endometrium, the expression of these genes is induced by progesterone and stimulated by IFNT and prostaglandins from the conceptus. This suggests a systemic response to pregnancy, where PBMCs may reflect endometrial activity or play an active role in immune modulation via localized cortisol synthesis. Rahmani *x* Barki crossbred exhibited the most pronounced upregulation, which may point to a heterosis effect enhancing the sensitivity of pregnancy recognition pathways, a finding that warrants further genetic investigation. This differential regulation, in which *HSD11B-1* facilitates cortisol production while *HSD11B-2* diminishes its effects, underscores the intricate hormonal balance necessary for a successful pregnancy.

A comparison of the expression trajectories of both genes reveals their distinct yet complementary roles. The expression of *HSD11B-1* exhibited a consistent increase from the post-implantation period through mid-gestation, underscoring its fundamental role in maintaining a cortisol-rich environment necessary for fetal development and maternal adaptation. In contrast, *HSD11B-2* expression displayed a different pattern; while also elevated post-implantation, it peaked earlier and began a significant decline during mid-gestation and prepartum luteolysis. This suggests that while *HSD11B-2* is essential initially for modulating glucocorticoid levels and protecting the conceptus, its regulatory role becomes less critical as parturition approaches. The sustained elevation of *HSD11B-1*, paired with the decline of *HSD11B-2*, points to a mechanism that ensures local cortisol availability increases toward term, which is crucial for initiating the parturition process. These differing trajectories highlight the sophisticated hormonal regulation that underpins successful gestation in sheep. The decline in expression of both genes during prepartum luteolysis aligns with the necessary hormonal shift from pregnancy maintenance to parturition initiation. This pattern is consistent with the known decline in progesterone and increased cortisol activity required for labour [39–41]. The present findings indicate that PBMC gene expression reflects this crucial terminal transition.

The robust expression of these genes in PBMCs presents a compelling opportunity for developing a novel, non-invasive early pregnancy test. A blood-based assay detecting *HSD11B-1* RNA as early as day 12 could transform reproductive management in sheep by allowing producers to identify non-pregnant ewes sooner, thereby reducing feeding costs and enabling rapid re-breeding. This approach would be less costly and less technically demanding than serial ultrasonography.

Our results are strongly supported by existing literature on the critical role of these enzymes in pregnancy. Brooks et al. [54]. demonstrated the importance of *HSD11B-1* for conceptus growth and elongation in ruminants, a finding reinforced by studies using specific inhibitors [55]. The concept that PBMCs can serve as a window into pregnancy status is further bolstered by work from Haq et al. [48]. and Yang et al. [56]., who reported upregulation of other pregnancy-related genes in blood cells. Furthermore, the distinct temporal roles we observed with *HSD11B-2* elevated post-implantation and *HSD11B-1* dominating mid-gestation closely mirror the findings of Pereira et al. [28]. in canine species, suggesting a potentially conserved mechanistic pathway across mammals.

Despite these promising insights, the study’s focus on five specific breeds may limit the immediate generalizability of the results. Future research should validate these biomarkers across a wider genetic and environmental spectrum. Furthermore, the precise molecular mechanisms triggering this PBMC response, including the specific transcription factors and signalling pathways involved, remain to be fully elucidated [23, 57–59].

The current points toward a viable commercial blood test for early pregnancy detection in sheep. The present study used pooled samples to robustly identify *HSD11B-1* and *HSD11B-2* as biomarkers. To turn this finding into a practical tool, future work must optimize RNA extraction from small blood volumes, establish clear diagnostic thresholds, and adapt the protocol for portable technology. A cost-benefit analysis indicates the test is economically viable, as the savings from early culling of non-pregnant ewes should significantly exceed the per-test cost. Successfully developing this test would offer a powerful new tool for precision reproductive management, improving both efficiency and sustainability in sheep farming.

## Conclusions

This study demonstrates that the genes *HSD11B-1* and *HSD11B-2* are significantly upregulated in the blood of pregnant sheep as early as 12 days after mating, highlighting their strong potential as biomarkers for early pregnancy detection and reproductive physiology across diverse breeds. Rahmani *x* Barki crossbred showed the most pronounced response, suggesting a genetic advantage in pregnancy recognition pathways. The distinct expression patterns of the two genes with *HSD11B-1* promoting a cortisol-rich environment for pregnancy maintenance and *HSD11B-2* modulating it for fetal protection reveal a sophisticated hormonal balance essential for gestation. These findings provide a foundation for developing a practical, blood-based test to identify pregnant ewes earlier than current methods allow. Such a tool could significantly improve sheep breeding management by enabling timely decisions that enhance flock productivity and welfare. Further research is warranted to translate these findings into commercial applications and explore the genetic mechanisms behind the breed-specific responses we observed.

### Data Availability

All data generated or analyzed during this study are included in this manuscript and its information files; Supplementary File (1): Supplementary Figures.

## Supplementary Information

Supplementary Material 1

## Data Availability

All data generated or analysed during this study are included in this manuscript and its information files; Supplementary File (1): Supplementary Figures.

## Abbreviations

qRT-PCR: Quantitative Real-Time Polymerase Chain Reaction
*HSD11B-1*: Hydroxysteroid Dehydrogenase 11 Beta 1
*HSD11B-2*: Hydroxysteroid Dehydrogenase 11 Beta 2
PBMCs: Peripheral Blood Mononuclear Cells
ewes: Female sheep
rams: Male sheep
kg: Kilograms
*p*: Probability value (statistical significance)
IFNT: Interferon Tau
CR: Control/Randomly selected ewes that were not allowed to mate
PRG: Pregnant
NPRG: Non-Pregnant
RSA-ASA: Randomly Selected Animals at the Same Age
TEW: Randomly Selected Ewes Allowed to Mate with Rams
C/EBP: CCAAT/Enhancer Binding Protein
PPAR: Peroxisome Proliferator-Activated Receptor
HNF1: Hepatocyte Nuclear Factor 1
LXR: Liver X Receptor
CR: Rahmani x Barki crossbred
F2α: Prostaglandin F2 Alpha
GR: Glucocorticoid Receptor
PGR: Progesterone Receptor
