# Supplementary figures and images for: Association of relative expression of HSD11B-1 and HSD11B-2 with pregnancy status in sheep
Source: BMC Genomics. 2025 Nov 17;26:1046. doi: 10.1186/s12864-025-12186-5 (PMC12621397; doi:10.1186/s12864-025-12186-5)

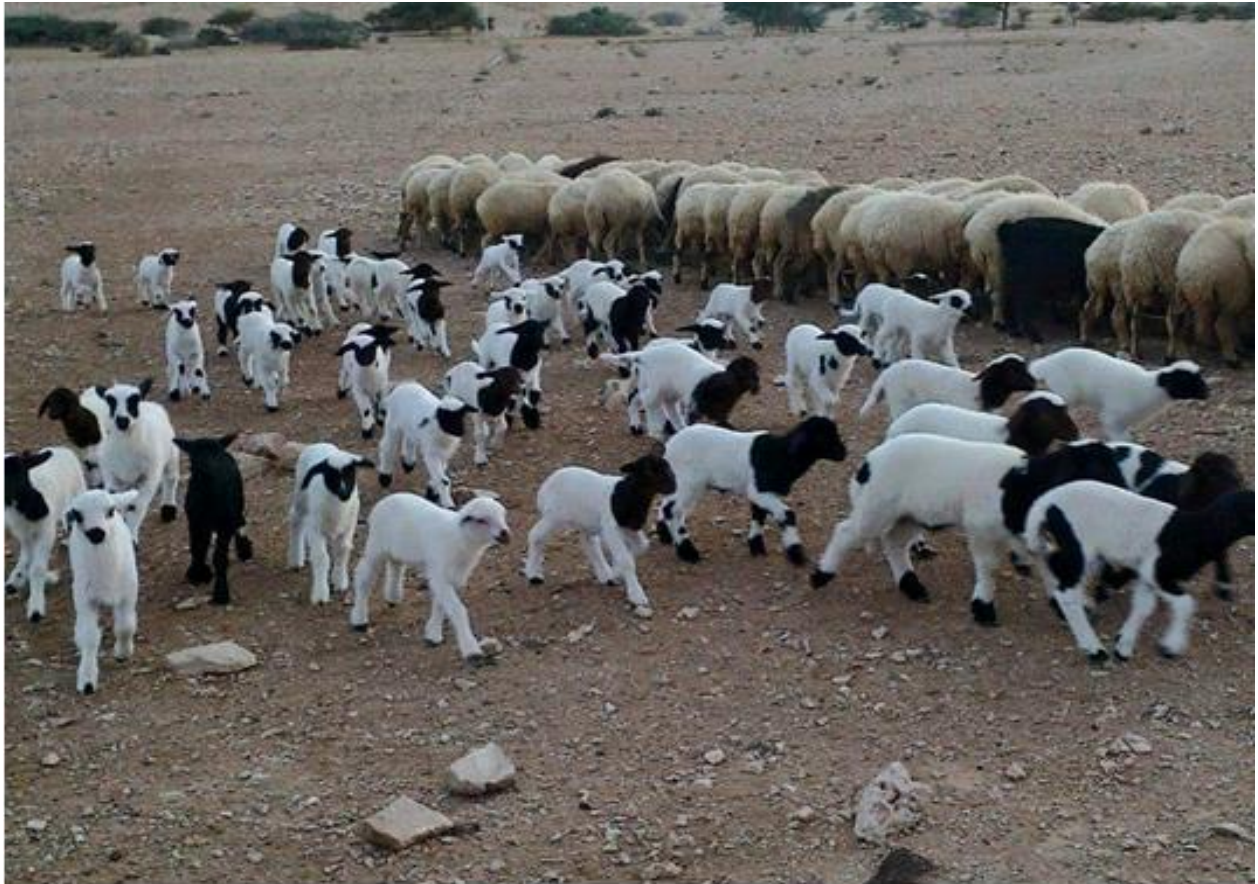

**Fig. S1.** The pure breeds and their progeny included in the present study seven months postpartum.

Supplement: Supplementary file 1 — Supplementary Material 1 [file 12864_2025_12186_MOESM1_ESM.pdf]
